# Hedgehog pathway activity in the LADY prostate tumor model

**DOI:** 10.1186/1476-4598-6-19

**Published:** 2007-03-07

**Authors:** Jerry Gipp, Guangyu Gu, Curtis Crylen, Susan Kasper, Wade Bushman

**Affiliations:** 1 University of Wisconsin Medical School, Department of Surgery, Division of Urology, 600 Highland Ave., K6-562 Clinical Sciences Center, Madison, WI 53792, USA; 2 Vanderbilt University Medical Center, Department of Urologic Surgery, AA-1315 MCN, 1161 21^st ^Avenue South, Nashville, TN 37232, USA

## Abstract

**Background:**

Robust Hedgehog (Hh) signaling has been implicated as a common feature of human prostate cancer and an important stimulus of tumor growth. The role of Hh signaling has been studied in several xenograft tumor models, however, the role of Hh in tumor development in a transgenic prostate cancer model has never been examined.

**Results:**

We analyzed expression of Hh pathway components and conserved Hh target genes along with progenitor cell markers and selected markers of epithelial differentiation during tumor development in the LADY transgenic mouse model. Tumor development was associated with a selective increase in Ihh expression. In contrast Shh expression was decreased. Expression of the Hh target Patched (Ptc) was significantly decreased while Gli1 expression was not significantly altered. A survey of other relevant genes revealed significant increases in expression of Notch-1 and Nestin together with decreased expression of HNF3a/FoxA1, NPDC-1 and probasin.

**Conclusion:**

Our study shows no evidence for a generalized increase in Hh signaling during tumor development in the LADY mouse. It does reveal a selective increase in Ihh expression that is associated with increased expression of progenitor cell markers and decreased expression of terminal differentiation markers. These data suggest that Ihh expression may be a feature of a progenitor cell population that is involved in tumor development.

## Background

*Sonic hedgehog (Shh)* is one of three mammalian hedgehog *(Hh)* genes *[Sonic hedgehog, Desert hedgehog, Indian hedgehog].* Each of the Hh genes encodes a secreted signaling peptide that binds to a membrane bound receptor (Ptc). Binding of Hh ligand to the Ptc receptor on the target cell initiates an intracellular signal transduction cascade that ultimately activates expression of Hh target genes through the activity of a family of Gli transcription factors [1]. Previous studies have identified Shh as an important regulator of prostate development [2-7]. Shh is expressed exclusively in the epithelium of the developing prostate. Expression is most abundant during prostate ductal budding and postnatal ductal morphogenesis and diminishes to a low level in the adult. Ihh is also expressed in the prostate epithelium. It is expressed at relatively lower levels in the developing prostate but in a distinctive pattern and, in contrast to Shh, its expression is maintained undiminished in the adult [8]. Shh expressed by the Urogenital Sinus (UGS) epithelium induces mesenchymal Hh target gene expression indicating a paracrine mechanism of action [2-5,7]. Paracrine signaling directly affects mesenchymal proliferation [3,7], but also influence epithelial proliferation and differentiation by paracrine feedback mechanisms [[4,7,9], manuscript in preparation]. Autocrine or juxtacrine signaling is a prominent feature of Hh actions in development [1] and several lines of evidence now suggest that autocrine signaling stimulates prostate epithelial proliferation [6,10].

Recently Hh signaling has been identified as an important factor in prostate cancer. Paracrine signaling from tumor cells has been shown to activate Hh target gene expression in the adjacent stroma and xenograft studies indicate that paracrine signaling can accelerate tumor growth [9]. In addition, evidence has been presented suggesting that autocrine signaling may also occur, particularly in advanced prostate cancer, and directly drive tumor cell proliferation [11-13]. These studies have generated considerable interest in understanding the role of Hh signaling in prostatic neoplasia. We report here the first characterization of Hh signaling in a transgenic mouse model of prostate cancer.

The LADY tumor is a transgenic mouse model of prostate cancer in which the large T antigen gene containing the deletion mutation d1 2005 (removing expression of small t antigen) is driven from the prostate specific probasin promoter [14]. As compared to the TRAMP mouse model, tumors in LADY transgenic mice (Line 12T-7f) develop and progress through epithelial hyperplasia, reactive stromal hyperplasia, dysplasia and mouse intraepithelial neoplasia (mPIN) similar to that observed in human high grade PIN. Transgene expression is under androgen control since the probasin promoter is developmentally regulated by androgens [14]. Thus, changes in epithelial cell architecture occur in all prostatic lobes as early as four weeks of age and are identified as clusters of cells containing elongated, hyperchromatic nuclei interspersed among normal epithelial cells. Reactive stromal proliferation occurs in parallel with epithelial cell transformation. These changes are complete by seven to eight weeks and the resulting tumors progress from lesions resembling human low-grade PIN with epithelial stratification and mild nuclear atypia to HGPIN with marked nuclear atypia by 15 weeks of age. Although rapid tumor growth precludes maintaining these mice much beyond four months, micro-invasion and metastases are rarely seen.

## Results

**Figure 1:**
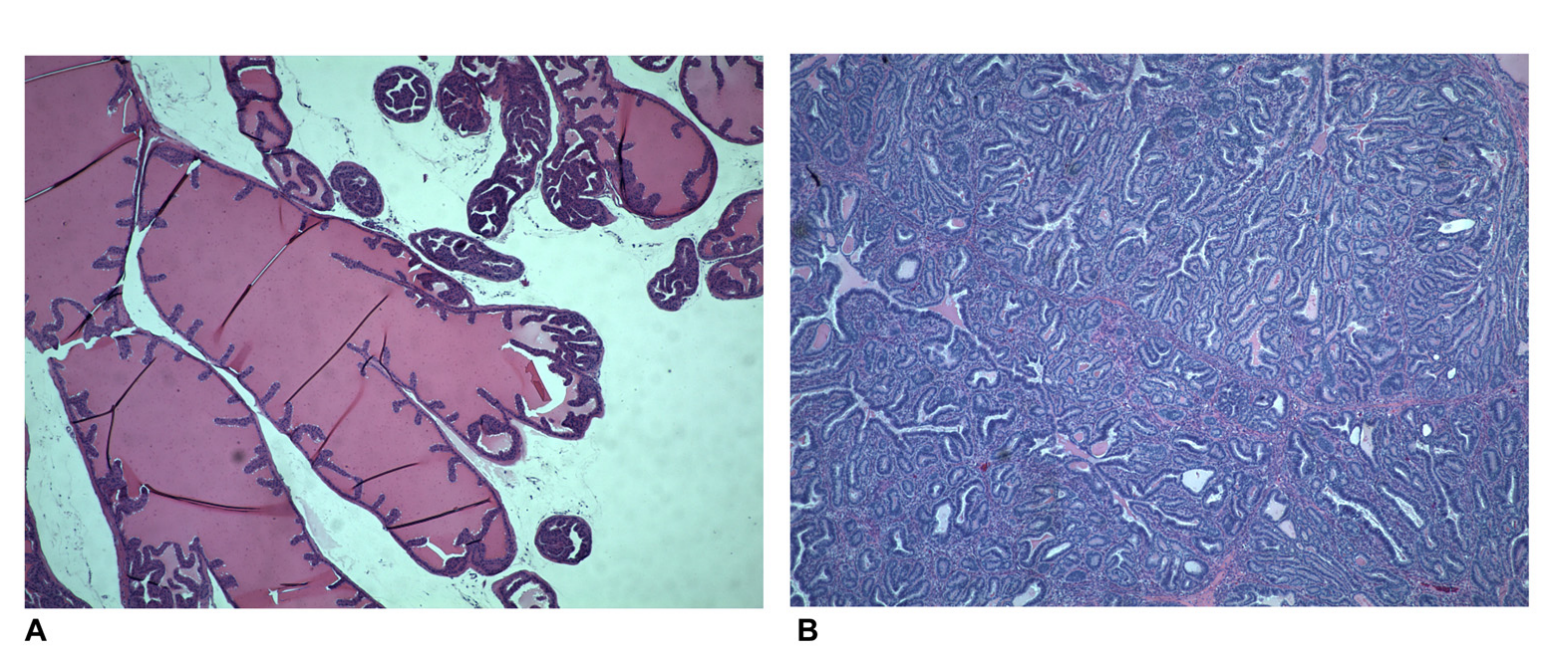
**Histological comparison of control CD-1 and LADY tumor**. A: H&E staining of the CG from normal litter mate B: H&E staining of the CG from a LADY tumor at 16 weeks of age.

The time-course of tumor development in the 12T-7f subline of the LADY transgenic mouse has previously been described [14]. We performed an analysis of Hh pathway gene expression in this subline, collecting tissues from controls and LADY mice at three, six, nine, 12 and 16 weeks. When mice were sacrificed at 16 weeks, there was noted to be massive enlargement of the coagulating gland (CG) of the LADY mice as compared to the controls and histologic examination confirmed the presence of diffuse hyperplasia, stromal reaction and in situ carcinoma as previously described (Fig. 1). To examine expression of the Hh pathway genes during tumor development, we examined gene expression in the CG at six and 16 weeks, time points corresponding to an early post-pubertal stage and to the adult stage of fully developed in situ carcinoma, respectively.

**Figure 2:**
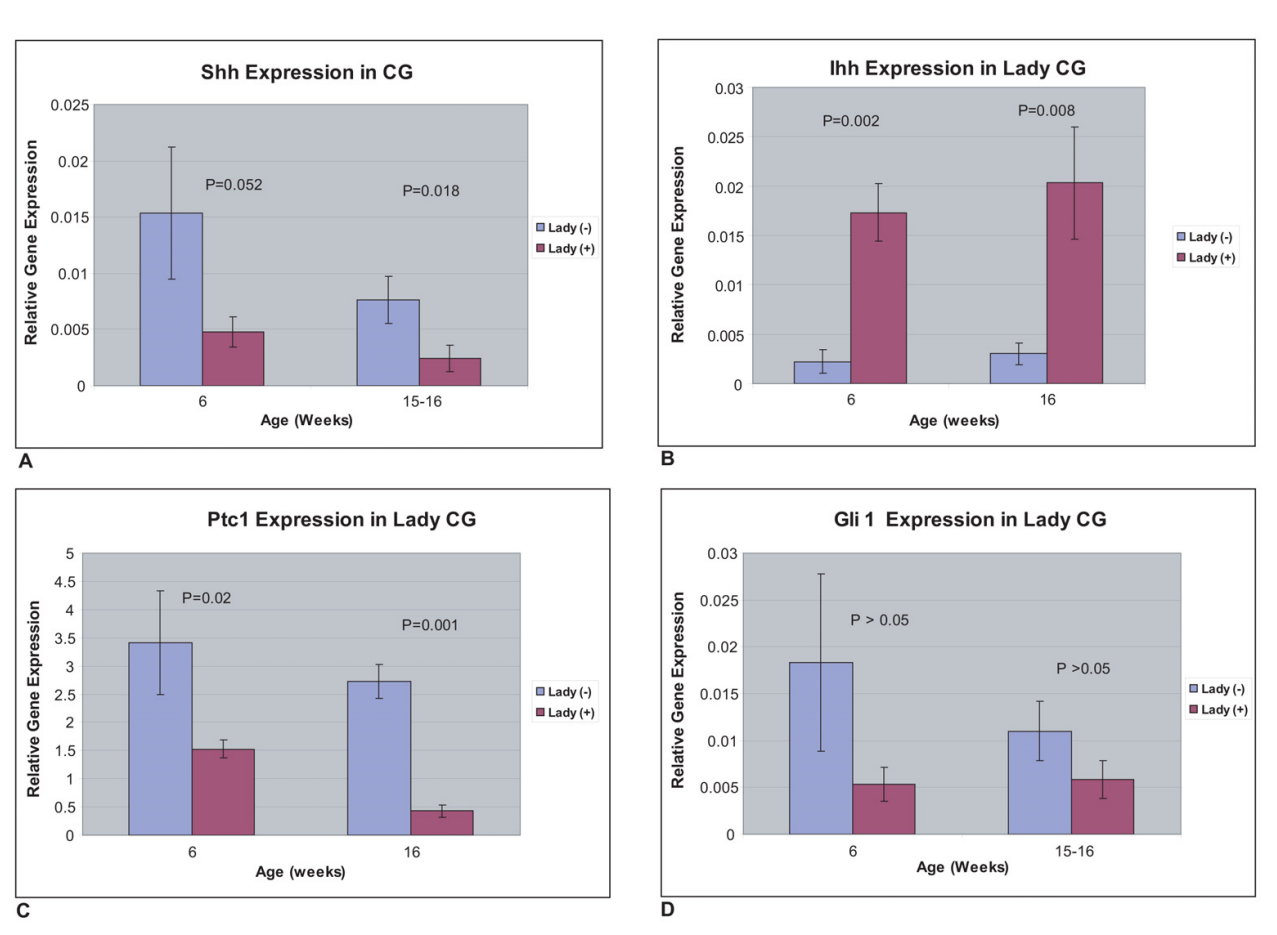
**Age dependent changes in Hh pathway gene expression**. Gene expression at six and 16 weeks of age in the CG of control CD-1 and LADY positive mice A: Shh, B: Ihh, C: Ptc1, D: Gli1.

Shh expression progressively diminished with age in both LADY and wild type control mice, but Shh expression trended lower at six weeks and was significantly lower at 16 weeks in the LADY mice. In contrast, Ihh expression was significantly increased in the LADY mice at both six and 16 weeks as compared to the WT controls (Fig. 2). To determine the net effect on Hh signaling, we examined the expression of the Shh receptor Ptc and the transcription factor Gli1, direct Hh target genes whose expression serves as a measure of Hh pathway activation. Ptc expression was significantly lower in the CG of the LADY mice than in the WT controls at all time points examined (three, six, nine, 12 and 16 weeks). The differences at six and 16 weeks are shown (Fig. 2). Similarly, Gli1 expression was also lower in the CG of the LADY mice although the differences did not reach statistical significance. Neither Gli2, Gli3 nor the Hh signaling pathway gene Smo exhibited significantly different expression at either six or 16 weeks (Fig. 2 and data not shown). These data indicate an overall decrease in the level of Hh signaling in the LADY tumor. However, the dichotomous changes in Hh ligand expression suggest the possibility of selective activation of Hh signaling within a particular niche. Accordingly, we examined the expression of several tissue-specific Hh target genes. IGFBP-6 is a target of Hh signaling during fetal prostate development and IGFBP-3 expression is activated by Hh treatment of the UGSM-2 cell line [[15,16], unpublished observations]. IGFBP-6 expression was decreased at both six and 16 weeks in the LADY mouse (Fig. 2) while expression of IGFBP-3 was unchanged (data not shown).

**Figure 3:**
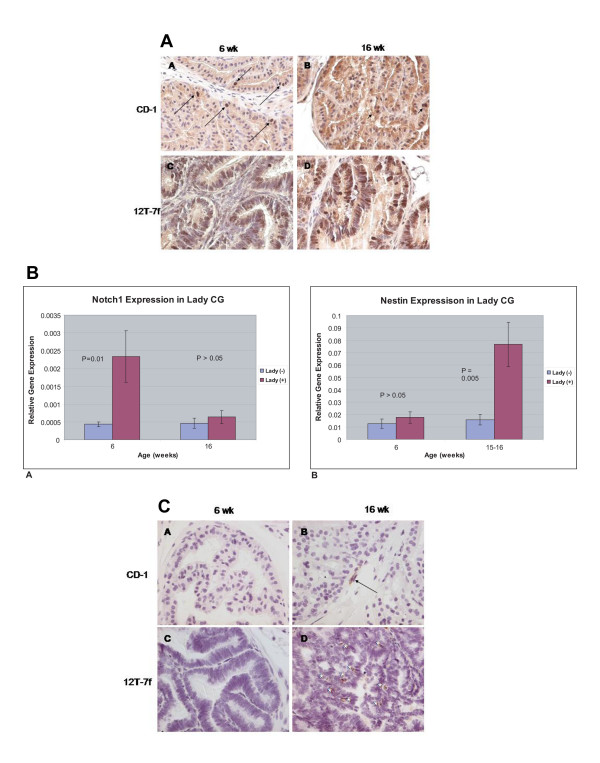
**Proliferation and associated gene expression in control CD-1 and LADY CG**. 3A: Panels A and B: Ki67 staining in six and 16 week CG was limited to a few scattered epithelial cells (A, arrow; B, arrowheads). Panels C and D: Greater than 50% of epithelial cells stained positive for Ki67. 3B: Proliferation associated gene expression changes at six and 16 weeks in control CD-1 and LADY CG. Panel A: Notch1, B: Nestin. 3C: Nestin staining at six and 16 week in Control and LADY CG Panels A and B: Little to no staining at six weeks with a few cells detected at 16 weeks in control CD-1 CG (Arrow = basal-like cell). Panels C and D: Increased nestin expression is observed in 16 week prostate tumor. Asteric = epithelial cells.

**Figure 4:**
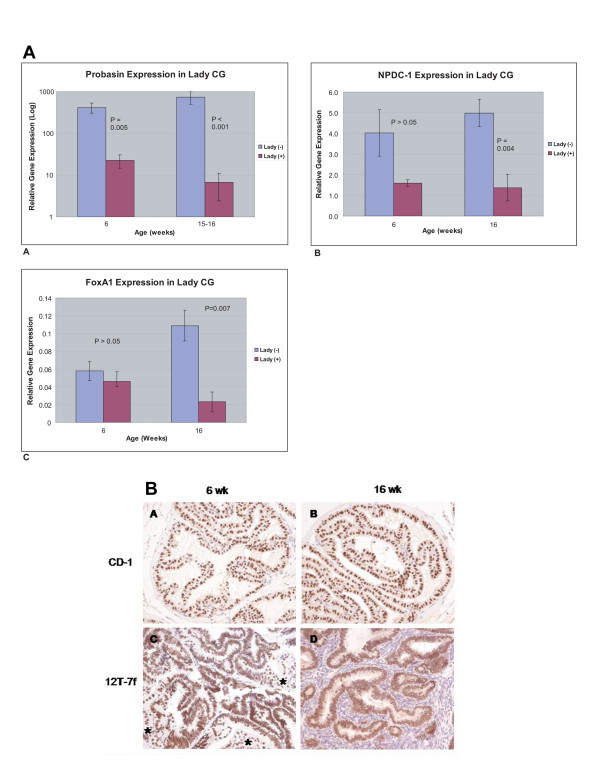
**Differention associated gene expression changes in LADY tumor development**. 4A: Differentiation gene expression analysis in control CD-1 and LADY CG at six6 and 16 weeks of age A: Probasin, B: NPDC-1, C: FoxA1. 4B: Foxa1 staining in Control and LADY CG. Panels A and B: FoxA1 protein is detected in the epithelial cells of six and 16 week CG. Panels C and D: Dysplastic epithelial cells continue to express high levels of Foxa1, asteric denotes areas of normal prostatic tissue.

To examine what changes in epithelial proliferation and differentiation accompany tumor development in the LADY mouse, we compared epithelial proliferation and differentiation at six and 16 weeks (Fig. 3). In the WT mice, Ki67 staining was detected in an occasional nucleus. In contrast, most of the nuclei in the LADY mice stained positive for Ki67 at six weeks and this rapid rate of proliferation was maintained at 16-week tumors. Notch-1 has been identified as a critical regulator of epithelial proliferation and differentiation during prostate development [17]. Notch-1 expression increased significantly in the six-week developing tumor, but not in the established 16-week tumor, suggesting an early role in the dynamics of epithelial proliferation and differentiation prior to the appearance of histologic changes. At 16 weeks, there was significantly increased expression of mRNA for the stem cell marker Nestin in the tumor and immunohistochemical staining localized the increased expression to the basal layer of the epithelium. FoxA1 and FoxA2 are important regulators of epithelial proliferation and differentiation during prostate development that exhibit altered patterns of expression in human prostate cancer [18]. Quantitative RT-PCR and immunohistochemical staining showed no change in the expression of FoxA2 (data not shown). Quantitative RT-PCR showed diminished FoxA1 mRNA expression that contrasted with increased staining for FoxA1 protein in the LADY tumor (Fig. 4). Expression of the differentiation markers Npdc-1 and probasin were significantly decreased in the tumor, suggesting an inhibition of luminal cell differentiation.

## Discussion

Several groups have examined the expression of Hh ligands and Hh signaling activity in human prostate cancer with inconsistent findings. We first reported that robust Shh expression and Gli1 expression was characteristic of both the normal adult human prostate as well as benign prostatic hyperplasia and prostate cancer [9]. Karhadkar [11] reported that Shh and Ihh were both expressed in localized prostate cancer and benign tissue but that the Hh target genes Ptc and Gli1 were expressed only in metastatic tumors. Sanchez et al [12] reported findings suggesting a basal level of Shh, Ptc and Gli1 expression in benign tissue that is variably increased in cancer while Sheng et al [13]observed increased Ptc expression that was attributable in part to mutations that dysregulate Hh signal transduction. These studies have left unresolved the role of Hh signaling in tumor development and progression and for this reason an examination of the Hh pathway in transgenic tumor models may be instructive. The data reported here clearly shows that a net increase in Hh signaling, as measured by expression of the conserved Hh target genes Ptc and Gli1, is not a feature of tumor development in the LADY mouse.

An unexpected and provocative finding was that a net decrease in Hh pathway activity during tumor development, as measured by Ptc expression, actually embraced divergent changes in expression of the two Hh ligands. Shh expression was decreased while Ihh expression was dramatically increased. These changes were evident as early as three weeks of age (data not shown). Shh and Ihh are highly related ligands which show functional redundancy in their ability to activate the Hh signal transduction pathway, but Shh and Ihh are expressed in unique patterns during prostate development. Whereas Shh expression localizes to the tips of the developing ducts, Ihh expression localizes to the epithelium of the urethral lumen and the origin of the developing ducts. Shh is expressed abundantly during prostate development and gradually diminishes to a low level in the adult prostate [4]. In contrast, lower level Ihh expression is maintained at a fairly constant level throughout development and in adulthood [8]. We have observed two other circumstances where prostate Ihh expression is increased. The first is during prostate development in the Shh transgenic null, where Hh signaling is maintained by a significant increase in Ihh expression [8]. The second is during castration-induced involution of the VP, where there is a marked increase in Ihh expression without a comparable increase in Shh expression (unpublished observations). Taken together, these findings suggest that Shh and Ihh are differentially regulated and, while exhibiting some degree of functionally redundancy, may normally play different roles in prostate growth regulation. Indeed, studies of Hh signaling in prostate development suggest that Hh signaling exerts a variety of effects, including stimulation of progenitor cell proliferation, regulating ductal epithelial proliferation and differentiation and ductal morphogenesis through a combination of autocrine and paracrine signaling [19]. The respective roles of Shh and Ihh in these effects remains to be elucidated, but we have been intrigued to discover that the domain of Ihh expression coincides with the regions where tissue specific stem cells appear to be present in relative abundance [20]. Our finding that Ihh expression is increased in response to castration suggests that Ihh is expressed by a progenitor cell population and we speculate that increased Ihh expression in the LADY tumor results from an expansion of that progenitor cell population during tumor development.

Tumor development in the LADY mouse is associated with an increased rate of epithelial proliferation at both six and 16 weeks, increased expression of progenitor cell markers (Notch-1, Nestin) and decreased expression of terminal differentiation markers (Npdc-1 and probasin). These findings are consistent with an expansion of progenitor and/or transit amplifying cell populations. FoxA proteins belong to the forkhead box a (FoxA) superfamily of transcription factors and are expressed in endodermally-derived tissues [21]. FoxA1 immunoreactivity is found in epithelial cells of both the developing and adult prostate [21]. We found that mRNA levels deceased significantly in the LADY tumor but that staining for FoxA1 protein was undiminished. Possible mechanisms for these findings would include increased rates of mRNA turnover or stabilization of FoxA1 protein stabilization in cells that do not udergo terminal differention. FoxA2 expression is prominent in developing bud epithelium during prostate development but localizes to basal epithelial cells of the adult periurethral ducts [21]. FoxA2 has been detected in neuroendocrine small cell carcinomas and high Gleason grade adenocarcinomas [18], but FoxA2 mRNA expression was not increased in the LADY tumors,

## Conclusion

There is no net increase in Hh signaling during tumor development in the LADY prostate cancer model. However, there is a selective and marked increase in Ihh expression even as Shh expression is decreased. Tumor development is associated with increased epithelial proliferation, increased expression on progenitor cell markers and decreased expression of terminal differentiation markers. Ihh expression may be associated with a progenitor cell population that is expanded during tumor development.

## Methods

### Animals

LADY mice, strain 12T-7f in a CD-1 background, were used for this study. Animals were bred, raised and sacrificed according to University of Wisconsin animal care and use guidelines. Prostate specific lobes were harvested at various times and either fixed in 10% formalin for sectioning or flash frozen for RNA isolation.

### RNA isolation and Real Time-PCR

RNA was isolated using Qiagen RNeasy kit with on column DNase treatment as per manufacturer's directions. Following reverse transcription by standard protocols, gene expression was quantitated by real time PCR using Power SYBR Green PCR master mix (Applied Biosystems) on a BioRad iCycler. Expression was normalized to the internal control gene glyceraldehydes-3-phosphate dehydrogenase (GAPDH) for each gene assayed. Gene specific primer sequences are as follows:GAPDH: 5'-AGCCTCGTCCCGTAGACAAAAT-3' and 5'-CCGTGAGTG GAGTCATACTGGA-3', Shh: 5'-AATGCC TTGGCCATCTCTGT-3' and 5'GCTCGACCCTCATAGTGTAGAGACT-3', Ihh: 5'-CAGCTCACCCCCAACTACAA-3' and 5'-GAGCTCACCCCCAACTACAA-3', Ptc1: 5'-CTCTGGAGCAGATTTCCAAGG-3' and 5'-TGCCGCAGTTCTTTTGAATG-3', Gli1: 5'-GGAAGTCCTATTCACGCCTTGA-3' and 5'CAACCTTCTTGCTCACACATG TAAG-3', Igfbp-6: 5'-AGCTCCAGACTGAGGTCTTCC-3' and 5'-GAACGACACTGCTGCTTGC-3', P21: 5'-TTGCACTCTGGTGTCTGAGC-3' and 5'-TCTGCGCTTGGAGTGATAGA-3', Notch1: 5'-ACCCACTCTGTCTCCCACAC-3' and 5'-GCTTCCTTGCTACCACAAGC'-3', Nestin: 5'-GGACAGGACCAAGAGGAACA-3' and 5'-TCTGGATCCACCTTTTCTGG-3', Probasin: 5'-TCATCCTCCTGCTCACACTG-3' and 5'-AAGGCCCGTCAATCTTCTTT-3', NPDC-1: 5'-CCTCCGATGAGGAAAATGAA-3' and 5'-CGTGGAATGGTCAAACAGTG-3', FoxA1: 5'-CTCCTTATGGCGCTACCTTG-3' and 5'-AGCACGGGTCTGGAATACAC-3'.

### Immunohistochemical analysis

Tissues were fixed in 10% buffered formalin overnight, followed by transfer to 50% alcohol. The paraffin-embedded tissues were sectioned (5 μm). Sections were deparaffinized and rehydrated in ethanol solutions. For FoxA1 (goat anti-FoxA1, Santa Cruz) staining, after antigen unmasking by boiling in 10 mM sodium citrate buffer (pH 6.0) for 20 min, the sections were treated with 3% hydrogen peroxide for 5 min. The following detection and visualization procedures were performed according to manufacturer's protocol (Vector Laboratories). For Ki67 staining was done at Vanderbilt Immunohistochemistry Core/Lab, the sections were rehydrated and placed in heated Target Retrieval Solution (Labvision, Fremont, CA) for 20 min. Endogenous peroxidase was neutralized with 0.03% hydrogen peroxide followed by a casein-based protein block (DakoCytomation, Carpinteria, CA) to minimize nonspecific staining. The sections were incubated with rabbit anti- Ki-67 (Vector Laboratories, Burlingame, CA) for 30 min. The Dako Envision+ HRP/DAB System (DakoCytomation) was used to produce localized, visible staining. Negative control slides were performed without primary antibodies.

### Statistical Methods

Two-Way Analysis of Variance (ANOVA) was used to compare expression levels as a function of age (six vs. 16 weeks), LADY gene [(-) vs (+)] and their interaction. This was done separately for each of the proteins (Shh, Ihh, Gli 1, Ptc 1, probasin, p21, nestin, NPDC-1, IGFBP-6). The tenability of the assumptions of ANOVA was assessed with residual plots. If the assumptions of ANOVA seemed to be violated, transformations of the response were considered. If a term in the model was statistically significant, pair-wise comparisons were obtained and examined for significance; this corresponds to Fisher's protected Least Significant Difference (LSD) procedure. P < 0.05 was the criterion for statistical significance. The analyses were performed with Proc GLM, SAS v.9.1 statistical software (SAS, Cary, NC). Results: The natural logarithms of the expression levels were log-transformed prior to ANOVA in order to homogenize the variances and better meet the assumptions of ANOVA.

## Competing interests

The author(s) declare that they have no competing interests.

## Authors' contributions

JG was responsible for the gene expression studies and participated in the preparation of this manuscript. GG and SK carried out the IHC staining and interpretation of data. CC was involved in discussions of these studies and drafted the manuscript. WB conceived the study, participated in its design and coordinated the final draft.

## References

[B1] Ingham PW, McMahon AP (2001). Hedgehog signaling in animal development: paradigms and principles. Genes Dev.

[B2] Berman DM, Desai N, Wang X, Karhadkar SS, Reynon M, Abate-Shen C, Beachy PA, Shen MM (2004). Roles for Hedgehog signaling in androgen production and prostate ductal morphogenesis. Dev Biol.

[B3] Freestone SH, Marker P, Grace OC, Tomlinson DC, Cunha GR, Harnden P, Thomson AA (2003). Sonic hedgehog regulates prostatic growth and epithelial differentiation. Dev Biol.

[B4] Lamm ML, Catbagan WS, Laciak RJ, Barnett DH, Hebner CM, Gaffield W, Walterhouse D, Iannaccone P, Bushman W (2002). Sonic hedgehog activates mesenchymal Gli1 expression during prostate ductal bud formation. Dev Biol.

[B5] Podlasek CA, Barnett DH, Clemens JQ, Bak PM, Bushman W (1999). Prostate development requires Sonic hedgehog expressed by the urogenital sinus epithelium. Dev Biol.

[B6] Pu Y, Huang L, Prins GS (2004). Sonic hedgehog-patched Gli signaling in the developing rat prostate gland: lobe-specific suppression by neonatal estrogens reduces ductal growth and branching. Dev Biol.

[B7] Wang BE, Shou J, Ross S, Koeppen H, De Sauvage FJ, Gao WQ (2003). Inhibition of epithelial ductal branching in the prostate by sonic hedgehog is indirectly mediated by stromal cells. J Biol Chem.

[B8] Doles J, Cook C, Shi X, Valosky J, Lipinski R, Bushman W (2006). Functional compensation in Hedgehog signaling during mouse prostate development. Dev Biol.

[B9] Gao N, Ishii K, Mirosevich J, Kuwajima S, Oppenheimer SR, Roberts RL, Jiang M, Yu X, Shappell SB, Caprioli RM, Stoffel M, Hayward SW, Matusik RJ (2005). Forkhead box A1 regulates prostate ductal morphogenesis and promotes epithelial cell maturation. Development.

[B10] Fan L, Pepicelli CV, Dibble CC, Catbagan W, Zarycki JL, Laciak R, Gipp J, Shaw A, Lamm ML, Munoz A, Lipinski R, Thrasher JB, Bushman W (2004). Hedgehog signaling promotes prostate xenograft tumor growth. Endocrinology.

[B11] Karhadkar SS, Bova GS, Abdallah N, Dhara S, Gardner D, Maitra A, Isaacs JT, Berman DM, Beachy PA (2004). Hedgehog signalling in prostate regeneration, neoplasia and metastasis. Nature.

[B12] Sanchez P, Hernandez AM, Stecca B, Kahler AJ, DeGueme AM, Barrett A, Beyna M, Datta MW, Datta S, Ruiz i Altaba A (2004). Inhibition of prostate cancer proliferation by interference with SONIC HEDGEHOG-GLI1 signaling. Proc Natl Acad Sci U S A.

[B13] Sheng T, Li C, Zhang X, Chi S, He N, Chen K, McCormick F, Gatalica Z, Xie J (2004). Activation of the hedgehog pathway in advanced prostate cancer. Mol Cancer.

[B14] Kasper S, Sheppard PC, Yan Y, Pettigrew N, Borowsky AD, Prins GS, Dodd JG, Duckworth ML, Matusik RJ (1998). Development, progression, and androgen-dependence of prostate tumors in probasin-large T antigen transgenic mice: a model for prostate cancer. Lab Invest.

[B15] Lipinski RJ, Cook CH, Barnett DH, Gipp JJ, Peterson RE, Bushman W (2005). Sonic hedgehog signaling regulates the expression of insulin-like growth factor binding protein-6 during fetal prostate development. Dev Dyn.

[B16] Shaw A, Papadopoulos J, Johnson C, Bushman W (2006). Isolation and characterization of an immortalized mouse urogenital sinus mesenchyme cell line. Prostate.

[B17] Wang XD, Leow CC, Zha J, Tang Z, Modrusan Z, Radtke F, Aguet M, de Sauvage FJ, Gao WQ (2006). Notch signaling is required for normal prostatic epithelial cell proliferation and differentiation. Dev Biol.

[B18] Mirosevich J, Gao N, Gupta A, Shappell SB, Jove R, Matusik RJ (2006). Expression and role of Foxa proteins in prostate cancer. Prostate.

[B19] Shaw A, Bushman W (2007). Hedgehog Signaling in the Prostate. The Journal of Urology.

[B20] Goto K, Salm SN, Coetzee S, Xiong X, Burger PE, Shapiro E, Lepor H, Moscatelli D, Wilson EL (2006). Proximal prostatic stem cells are programmed to regenerate a proximal-distal ductal axis. Stem Cells.

[B21] Mirosevich J, Gao N, Matusik RJ (2005). Expression of Foxa transcription factors in the developing and adult murine prostate. Prostate.

